# Multi-Sensor Combined Measurement While Drilling Based on the Improved Adaptive Fading Square Root Unscented Kalman Filter

**DOI:** 10.3390/s20071897

**Published:** 2020-03-29

**Authors:** Yi Yang, Fei Li, Yi Gao, Yanhui Mao

**Affiliations:** School of Electronic Engineering, Xi’an Shiyou University, Xi’an 710065, China

**Keywords:** multi-sensor combined measurement, quaternion, unscented kalman filter, square root filter, adaptive fading factor

## Abstract

In the process of the attitude measurement for a steering drilling system, the measurement of the attitude parameters may be uncertain and unpredictable due to the influence of server vibration on bits. In order to eliminate the interference caused by vibration on the measurement and quickly obtain the accurate attitude parameters of the steering drilling tool, a new method for multi-sensor dynamic attitude combined measurement is presented. Firstly, by using a triaxial accelerometer and triaxial magnetometer measurement system, the nonlinear model based on the quaternion is established. Then, an improved adaptive fading square root unscented Kalman filter is proposed for eliminating the vibration disturbance signal. In this algorithm, the square root of the state covariance matrix is used to replace the covariance matrix in the classical unscented Kalman filter (UKF) to avoid the filter divergence caused by the negative definite state covariance matrix. The fading factor is introduced into UKF to adjust the filter gain in real-time and improve the adaptive ability of the algorithm to mutation state. Finally, the computational method of the fading factor is optimized to ensure the self-adaptability of the algorithm and reduce the computational complexity. The results of the laboratory test and the field-drilling data show that the proposed method can filter out the interference noise in the attitude measurement sensor effectively, improve the solution accuracy of attitude parameters of drilling tools in the case of abrupt changes in the measuring environment, and thus ensuring the dynamic stability of the well trajectory.

## 1. Introduction

With the continuous development of the petroleum industry and the increasing difficulty of oil and gas exploration and development, carrier space attitude measurement plays an increasingly prominent role in the field of the petroleum industry [1,2,3,4]. This means that the requirements of real-time, accuracy, continuous, and dynamic measurement of drilling tool attitude parameters (inclination, tool face angle, and azimuth angle) are higher and higher. In the process of drilling, due to the influence of the drilling string rotation, server vibration near bits, high temperature, high pressure, and a strong magnetic field, especially in the near vertical condition (when the inclination is less than 1°), if only using the three-axis accelerometer sensors, the three-axis magnetometer sensors or simply combining them in mechanical, still show significant errors for the attitude parameters of the guiding drilling tools [5,6]. At this time, using a multi-sensor combined measurement system to generate redundant signals is one of the research directions to realize dynamic measurement while drilling of attitude parameters.

There are two conventional multi-sensor combined systems for measurement while drilling (MWD) based on magnetometer sensors and gyroscope sensors, respectively. The system is called Gyroscope-based system, which uses gyro and accelerometer, as well as a magnetometer-based system using magnetometer sensors and accelerometer sensors because, in the drilling engineering, the accelerometer is always used in various measuring systems [4,7].

Continuous MWD is studied under laboratory conditions using a gyroscope-based system. Mahmoud et al. [7,8] and Jurkov et al. [9] proposed an advanced inclination and direction sensor package based on an inertial navigation system (INS). They verified the reliability of the algorithm through simulation, which used INS to achieve continuous MWD with high accuracy. The influences of vibration and temperature on MWD were also analyzed [10,11,12]. However, because of their insufficient consideration about the down-hole complex situations, severe vibration is a great challenge for the measurement accuracy and lifetime of the sensors. Additionally, an increasing temperature can cause drift error in the gyroscope. Zhang et al. [13] and Chen et al. [14] developed a MWD instrument based on the simplified inertial measurement unit. However, there are still the same limitations as shown in the above studies.

Xue et al. [15] developed a strap-down measurement system based on a magnetometer, with triaxial accelerometer sensors and triaxial magnetometer sensors installed near the bit. To achieve continuous measurement, a novel linear stochastic system was proposed and the Kalman filter (KF) was used to estimate the states. They proved that a continuous-survey system with a KF approach can improve measurement precision and reduce errors produced by drill string vibration. However, the bottom drilling tool shows complex nonlinear stochastic characteristics while rotating due to a combined effect of their randomly nonlinear moving state, such as vertical vibration, horizontal vibration, eddy, and sticky slip. Therefore, there will be an obvious model error between the linear stochastic system established in [15] and the field-drilling dynamic system. Unfortunately, KF is only suitable for multi-sensor linear stochastic systems [16,17]. For the multi-sensor nonlinear stochastic systems, KF cannot be used.

The unscented Kalman filter (UKF) is a promising filtering method to estimate the state of a nonlinear stochastic system [18,19]. This method can approximate the posterior mean and covariance of any Gaussian random variable in third-order accuracy by using unscented transformation (UT). It has advantages of high estimation accuracy, high convergence rate, and simple implementation compared to other nonlinear filtering methods [19,20]. Due to these merits, Xu et al. [21], Yang et al. [22], and Gao et al. [5] developed the multi-sensor measurement system, which is composed of triaxial accelerometer sensors and triaxial magnetometer sensors, and the nonlinear model of attitude measurement based on quaternion is established, as well as the UKF method which is used to filter the vibration interference signal. The experimental results show that the method is effective in multi-sensor combined MWD. 

According to the literature analysis above, the UKF algorithm can eliminate the adverse effect of vibration disturbance to a certain extent by iterative calculation of the filtering model for drilling tool attitude parameters based on quaternion. However, due to the inherent defects of UKF, there are two problems in the dynamic calculation of drilling tool attitude parameters: (1) In the transmitting process of a covariance matrix, due to rounding error and noise, it is easy to cause numerical calculation instability [23,24,25], especially the influence of bottom vibration and the fast rotation of the drill pipe, which will aggravate the adverse effect of noise on calculation stability, and even lead to UKF algorithm failure; and (2) the filter gain of classical UKF cannot be adjusted online, as well as a lack of adaptive ability [26,27,28]. When the multi-sensor measurement system is abnormal (affected by the complex environment, such as high temperature and pressure, strong magnetic field, and near-vertical position, which leads to unnatural transfer or mutation of the attitude parameters while drilling), it cannot effectively track the attitude parameters while drilling.

Based on the classical UKF algorithm, this paper proposes an improved adaptive fading square root UKF (IAFSR-UKF) algorithm, which can dynamically process the attitude measurement signal by a multi-sensor combined system. This method combines the numerical stability of a square root filter and the strong tracking ability of an adaptive fading filter to the system state mutation. It can further eliminate or weaken the adverse effect of noise signal for the MWD and thereby obtain the real-time and high-precision attitude parameters, thus ensure the dynamic stability of the well trajectory. This method is applied to the nonlinear filtering model of the magnetometer-based system to solve the dynamic attitude parameters. The simulation test was carried out by the vibration platform system and the field-drilling data of a well in western Sichuan, which verified the accuracy and real-time performance of the proposed IAFSR-UKF applied to the combined measurement system for the drilling tools attitude parameters.

## 2. Multi-Sensor Combination Measurement Based on the IAFSR-UKF

### 2.1. A nonlinear System Model of MWD Based on Quaternion

According to the installation principle of three-axis sensor in attitude measurement while drilling and the common space coordinate transformation method in the field of inertial navigation, quaternion was used to replace the attitude parameters of drilling tools in the space attitude measurement, and a nonlinear filtering model of downhole attitude measurement based on quaternion is established in this section.

**Figure 1:**
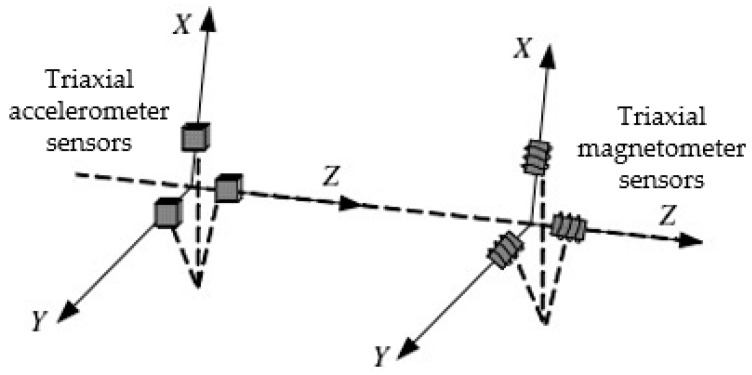
Installation diagram of triaxial measurement sensors for the measurement while drilling (MWD).

The multi-sensor measurement system was composed of three-axis accelerometer and three-axis magnetometer, as shown in Figure 1. Referring to the rotation coordinate transformation method in inertial navigation, the multi-sensor observation equation based on time-varying quaternion was established as follows.

(1) $$Y(t)={\left[\begin{array}{ccc} \begin{array}{ccc} \begin{array}{cc} m_{x} & m_{y} \end{array} & m_{z} & a_{x} \end{array} & a_{y} & a_{z} \end{array}\right]}^{T}=H\left[Q(t)\right]+{\left[\begin{array}{cccc} \begin{array}{ccc} n_{m_{x}} & n_{m_{y}} & n_{m_{z}} \end{array} & n_{a_{x}} & n_{a_{y}} & n_{a_{z}} \end{array}\right]}^{T},$$

where *m_x_*, *m_y_*, and *m_z_* are the components of the three-axis magnetometer sensors in the x, y, and z axes (*μ*T); *a_x_*, *a_y_*, and *a_z_* are the components of the three-axis accelerometer sensors in the x, y, and z axes (m/s^2^); *n_m_* and *n_a_* are the interference noise signals (*μ*T, m/s^2^) of magnetometer and accelerometer, respectively; ***Q***(*t*) is the value of quaternion *q*_0_, *q*_1_, *q*_2_, and *q*_3_ at time *t*, and ***H*** is the nonlinear observation matrix.

According to the theory of spatial coordinate rotation and the quaternion differential, the equation is as follows:

(2) $$Q^{\prime }=A\cdot Q.$$

The process equations expressed by quaternion are as follows:

(3) $${\left[\begin{array}{cccc} {q^{\prime }}_{0} & {q^{\prime }}_{1} & {q^{\prime }}_{2} & {q^{\prime }}_{3} \end{array}\right]}^{T}=A\cdot {\left[\begin{array}{cccc} q_{0} & q_{1} & q_{2} & q_{3} \end{array}\right]}^{T},$$

where

(4) $$A=\left[\begin{array}{cccc} 0 & -\omega_{x} & -\omega_{y} & -\omega_{z}\\ \omega_{x} & 0 & \omega_{z} & -\omega_{y}\\ \omega_{y} & -\omega_{z} & 0 & \omega_{x}\\ \omega_{z} & \omega_{y} & -\omega_{x} & 0 \end{array}\right].$$

According to the principle of gyro attitude measurement, the value of the three-axis angular velocity at time *t* can be obtained by the following formula:

(5) $$\omega (t)={\left[\begin{array}{ccc} \omega_{x} & \omega_{y} & \omega_{z} \end{array}\right]}^{T}=T(t)\cdot {\left[\begin{array}{ccc} \omega \cos\varphi & 0 & \omega \sin\varphi \end{array}\right]}^{T},$$

where ***ω*** is the earth’s rotation speed (rad/s); *ω*_x_, *ω*_y_, and *ω*_z_ are the components of the earth’s rotation speed on each axis in the XYZ coordinate system; ***T***(t) is the value of the three Euler rotation transformation matrix at time *t*; *ϕ* is the measured local latitude. 

According to the transformation relationship between three Euler angles and quaternion, the rotation matrix ***T*** which is represented by quaternion can be expressed as follows [21]:

(6) $$T=\left[\begin{array}{ccc} q_{0}^{2}+q_{1}^{2}-q_{2}^{2}-q_{3}^{2} & 2(q_{1}q_{2}+q_{0}q_{3}) & 2(q_{1}q_{3}-q_{0}q_{2})\\ 2(q_{1}q_{2}-q_{0}q_{3}) & q_{0}^{2}-q_{1}^{2}+q_{2}^{2}-q_{3}^{2} & 2(q_{2}q_{3}+q_{0}q_{1})\\ 2(q_{1}q_{3}+q_{0}q_{2}) & 2(q_{2}q_{3}-q_{0}q_{1}) & q_{0}^{2}-q_{1}^{2}-q_{2}^{2}+q_{3}^{2} \end{array}\right].$$

Assuming that the initial state of the state variable in the classical UKF is *Q*(0), the classical UKF solution steps can be found in [5].

### 2.2. IAFSR-UKF Based Multi-Sensor Combined Measurement

#### 2.2.1. The Description of Square Root Filter

The square root UKF uses the square root to replace the state covariance matrix in the filtering equation, and thereby ensure the non-negative quality of the covariance matrix and the numerical stability of the filtering algorithm. In addition, the square root UKF uses the square root ***S***(***SS***^T^ = ***P***) of the covariance matrix, and the Cholesky decomposition only requires a *n*^3^/6 calculation (*n* is the dimension of the state variable). Therefore, aiming at problem 1 of the UKF algorithm mentioned in the introduction, it can be solved with the help of square root filtering, which can be divided into the following two steps:

**Step 1:** QR Decomposition

Square root matrix ***S*** can be obtained by QR decomposition instead of Cholesky decomposition. For a matrix ***A***∈**R**^n×n^, we find an orthogonal matrix ***Q***∈**R**^n×n^ and an upper triangular matrix ***R***∈**R**^n×n^, so that ***A***^T^ = ***QR*** represents the return value of ***R*** in QR decomposition with *qr*(·). According to the theory of matrix analysis, the upper triangular matrix in ***R*** is ***S***^T^, ***S*** = *chol*(***P***), ***P*** = ***AA***^T^.

**Step 2:** Cholesky Factor Update

If ***S*** = *chol*(***P***), then square root matrix ***S*** is the Cholesky decomposition of matrix ***P***. Then, the successive update of Cholesky decomposition of matrix ***P*** ± ***v***^½^***uu***^T^ is recorded as ***S*** = *cholupdate*{***S***, ***u***, ±***v***}, where ***u*** is usually a column vector.

#### 2.2.2. The Description of Adaptive Fading Filter

The nonlinear system is defined as follows:

(7) $$\left\{ \begin{array}{l} X_{k}=f(X_{k-1},u_{k-1})+W_{k-1}\\ Z_{k-1}=h(X_{k-1})+V_{k-1} \end{array}\right.,$$

where ***W****_k_* and ***V****_k_* are uncorrelated Gaussian white noises.

The strong tracking filter could solve the nonlinear filtering problem of the above formula, and the sufficient condition for UKF to become a strong tracking filter is that it must satisfy the orthogonality principle. When the filter gain matrix ***K****_k_* is adjusted adaptively online, the following two conditions are met:

(8) $$E\left[\left(X_{k}-{\hat{X}}_{k}\right){\left(X_{k}-{\hat{X}}_{k}\right)}^{T}\right]=min,$$

(9) $$E\left(\varepsilon_{k}\varepsilon_{k+j}^{T}\right)=0,\;k=0,1,2,\cdots ;j=1,2,\cdots ,$$

where ***ε*** is the residual sequence of measured values.

The precondition of the above equation is that the residual sequences *ε_k_* must be orthogonal at any time. According to the principle of orthogonality, it is essentially to add a residual output sequence on the premise of the minimum variance performance index of state variable residual estimation. When the state estimation of the filter is abnormal, it can be represented by the mean value and amplitude of the output residual sequence. Strong tracking UKF can adjust the covariance matrix of the prediction error in real-time by increasing the fading factor, realize the online adjustment of filter gain, force (9) to hold, maintain the orthogonality of residual sequence, and achieve the purpose of strong tracking of the actual system state. Therefore, aiming at problem 2 of the UKF algorithm mentioned in the introduction, the strong tracking filter is introduced to solve it.

#### 2.2.3. The Design of IAFSR-UKF

According to the classical UKF, (8) has been already satisfied, and the method to determine the filter gain when (9) is satisfied is given in [27]. In this section, on the basis of the UKF algorithm, the adaptive fading filter and square root filter are combined to classical UKF, the determination method of the fading factor will be optimized, and finally, the IAFSR-UKF is presented. The main steps are as follows:

The system equations based on quaternion are discretized as follows:

(10) $$\left\{ \begin{array}{l} Q_{t+1}=(I+t_{s}A_{t})Q_{t}+w_{t}\\ Z_{t+1}=h(Q_{t})+v_{t} \end{array}\right.,$$

where ***Q****_t_* is the value of the quaternion at time *t*; ***I*** is the unit matrix; *t_s_* is the sampling period; ***w****_t_* and ***v****_t_* are the system noise and the sensor observation noise, respectively, meeting the requirements of ***w****_t_*~*N*(0, ***O****_t_*), ***v****_t_*~*N*(0, ***R****_t_*); ***h***(·) is the nonlinear observation matrix.

**Step 1:** Initialization

The initial state variable is a small random number and the initial variance matrix is a diagonal matrix.

**Step 2:** Sigma Point Update

Calculating 2*n* + 1 sample points at time *t* − 1 as follows:

(11) $$\left\{ \begin{array}{l} \chi_{t-1}^{0}=Q_{t-1}\\ \chi_{t-1}^{i}=Q_{t-1}+\gamma S_{t-1}\;i=1,2,\cdots ,n\\ \chi_{t-1}^{i}=Q_{t-1}-\gamma S_{t-1}\;i=n+1,n+2,\cdots ,2n \end{array}\right.,$$

where ***χ*** is the sigma point; ***S*** is the square root of the state covariance matrix; *γ* = (*n* + *λ*)^1/2^, *λ* = *α*^2^(*n* + *K*) − *n*. *α* is the scale factor, regulating the distribution distance of particles, generally between 0.001 and 1; *n* is the dimension of the system state variable; *K* is the third scale factor, generally taken as 0.

**Step 3:** Prediction Process

(12) $$\chi_{t/t-1}^{i}=(I+t_{s}A(t-1))\chi_{t-1}^{i},$$

(13) $$Q_{t/t-1}=\sum_{i=0}^{2n}W_{i}^{m}\chi_{t/t-1}^{i},$$

(14) $${\hat{S}}_{t/t-1}=qr[\sqrt{W_{1}^{c}}(\chi_{t/t-1}^{1:2n}-Q_{t/t-1}),\sqrt{O_{t-1}}],$$

(15) $$S_{t/t-1}=cholupdate({\hat{S}}_{t/t-1},\chi_{t/t-1}^{0}-Q_{t/t-1},W_{0}^{c}),$$

(16) $$\zeta_{t/t-1}^{i}=h(\chi_{t/t-1}^{i}),$$

(17) $$Z_{t/t-1}=\sum_{i=0}^{2n}W_{i}^{m}\zeta_{t/t-1}^{i}.$$

**Step 4:** Renewal Process

(18) $$P_{t/t-1}^{\left(XZ\right)}=\sum_{i=0}^{2n}W_{i}^{c}[\chi_{t/t-1}^{i}-Q_{t/t-1}]{\left[\zeta_{t/t-1}^{i}-Z_{t/t-1}\right]}^{T},$$

(19) $${\hat{S}}_{t/t-1}^{Z}=qr[\sqrt{W_{1}^{c}}(\zeta_{t/t-1}^{1:2n}-Z_{t/t-1}),\sqrt{R_{t}}],$$

(20) $$S_{t/t-1}^{Z}=\lambda_{t}\cdot cholupdate[{\hat{S}}_{t/t-1}^{Z},\zeta_{t/t-1}^{0}-Z_{t/t-1},W_{0}^{c}],$$

where ***P***^(*XZ*)^ is the cross-covariance matrix, ***S****^Z^* is the square root of the output covariance matrix, and *λ* is the fading factor.

In the above process, the weight coefficients of mean and covariance are respectively

(21) $$W_{i}^{m}=\left\{ \begin{array}{ll} \lambda /(n+\lambda ), & i=0\\ 1/2(n+\lambda ), & i\neq 0 \end{array}\right.,$$

(22) $$W_{i}^{c}=\left\{ \begin{array}{ll} \lambda /(n+\lambda )+(1+\beta -\alpha^{2}), & i=0\\ 1/2(n+\lambda ), & i\neq 0 \end{array}\right..$$

**Step 5:** Calculating the Fading Factor

Currently, the standard calculation method of the fading factor is a one-step algorithm [26], as shown below:

(23) $$\lambda_{t}=max\left\{1,tr(N_{t})/tr(M_{t})\right\},$$

where the symbol *tr*(·) represents matrix trace, and the expressions of ***M****_t_* and ***N****_t_* are as follows:

(24) $$\left\{ \begin{array}{l} M_{t}=H_{t}F_{t/t-1}P_{t/t-1}F_{t/t-1}^{T}H_{t}^{T}\\ N_{t}=C_{t}-H_{t}Q_{t}H_{t}^{T}-R_{t} \end{array}\right.,$$

where

(25) $$P_{t/t-1}=S_{t/t-1}S_{t/t-1}^{T},$$

(26) $$C_{t}=\left\{ \begin{array}{ll} Y_{t}Y_{t}^{T}, & t=1\\ \frac{\rho C_{t-1}+Y_{t}Y_{t}^{T}}{1+\rho }, & t\geq 2 \end{array}\right.,$$

(27) $$Y_{t}=Z_{t}-H_{t}X_{t/t-1},$$

(28) $$F_{t/t-1}=\frac{\partial A(X_{t})}{\partial X_{t}}\;H_{t}=\frac{\partial h(X_{t/t-1})}{\partial X_{t/t-1}},$$

where *ρ* is the forgetting factor; ***Z****_t_* is the measurement value of the attitude measurement sensors at the *t*-th data update; ***F****_t/t-1_* and ***H****_t_* are the Jacobi expansion of the state equation and the measurement equation, respectively.

**Step 6:** Calculating the Gain Matrix

(29) $$K_{t}={\left(P_{t/t-1}^{\left(XZ\right)}/(S_{t/t-1}^{Z}\right)}^{T}){\left(S_{t/t-1}^{Z}\right)}^{-1}.$$

**Step 7:** Status Update

(30) $$Q_{t}=Q_{t/t-1}+K_{t}(Z_{t}-Z_{t/t-1}),$$

(31) $$U=K_{t}S_{t/t-1}^{Z},$$

(32) $$S_{t}=cholupdate(S_{t/t-1},U,-1).$$

The results of the above filtering are applied to the attitude calculation of the drilling tool, and the filtered attitude angle of the drilling tool is obtained.

#### 2.2.4. The Improved Calculation Method for Fading Factor

From (28), it can be seen that the Jacobi matrix needs to be calculated when the fading factor *λ* is solved. For the nonlinear model of MWD based on quaternion, the calculation of the algorithm will be significantly increased by using three-axis accelerometer and three-axis magnetometer as the measurement sensors. Therefore, by studying the equivalent description of a strong tracking filter, this section gives an equivalent calculation method of the fading factor.

**Theorem** **1.** *It is assumed that the state covariance matrix, cross-covariance matrix, and output covariance matrix of UKF are respectively as follows:*

(33) $$P_{t/t-1}=E[(Q_{t}-Q_{t/t-1}){\left(Q_{t}-Q_{t/t-1}\right)}^{T}]=F_{t/t-1}P_{t-1}F_{t/t-1}^{T}+O_{t},$$

(34) $$P_{t/t-1}^{\left(ZZ\right)}=E[(Z_{t}-Z_{t/t-1}){\left(Z_{t}-Z_{t/t-1}\right)}^{T}]=H_{t}P_{t/t-1}H_{t}^{T}+R_{t},$$

(35) $$\begin{array}{ll} P_{t/t-1}^{\left(XZ\right)} & =E[(Q_{t}-Q_{t/t-1}){\left(Z_{t}-Z_{t/t-1}\right)}^{T}]=E[(Q_{t}-Q_{t/t-1}){\left(H_{t}(Q_{t}-Q_{t/t-1})\right)}^{T}]+E[(Q_{t}-Q_{t/t-1})v_{t}^{T}]\\ & =P_{t/t-1}H_{t}^{T}+E[(Q_{t}-Q_{t/t-1})v_{t}^{T}] \end{array}$$

*Then, the matrices **M**_t_ and **N**_t_ for solving the fading factor can be expressed by the following equations:*

(36) $$N_{t}=C_{t}-{\left(P_{t/t-1}^{\left(XZ\right)}\right)}^{T}{\left(S_{t/t-1}S_{t/t-1}^{T}\right)}^{-1}\cdot Q_{t}{\left(S_{t/t-1}S_{t/t-1}^{T}\right)}^{-1}P_{t/t-1}^{\left(XZ\right)}-R_{t},$$

(37) $$M_{t}={\left(P_{t/t-1}^{\left(XZ\right)}\right)}^{T}{\left(S_{t/t-1}S_{t/t-1}^{T}\right)}^{-1}\cdot (S_{t/t-1}S_{t/t-1}^{T}-Q_{t}){\left(S_{t/t-1}S_{t/t-1}^{T}\right)}^{-1}P_{t/t-1}^{\left(XZ\right)}.$$

**Proof.**  Since the matrix (***Q****_t_ − **Q**_t/t-1_*) and the noise matrix **v**_t_ are orthogonal, the (35) can be written as follows:

(38) $$P_{t/t-1}^{\left(XZ\right)}=P_{t/t-1}H_{t}^{T}.$$

Further, the (33) can be written as follows:

(39) $$F_{t/t-1}P_{t-1}F_{t/t-1}^{T}=P_{t/t-1}-Q_{t}.$$

Suppose ***Q****_t_* is a positive definite matrix, and according to the classical UKF, the inverse matrix of ***P****_t/t-1_* must exist, so (38) can be written as follows:

(40) $$H_{t}^{T}={\left(P_{t/t-1}^{\left(XZ\right)}\right)}^{T}{\left(P_{t/t-1}^{T}\right)}^{-1}.$$

In this case, the (25), (39), and (40) are substituted into (24), so the (36) and (37) are established.

The proof of Theorem 1 is completed. □

Thus, the fading factor can be calculated by the Formulas (23), (36), (37), (26), (27), and (40), (15) successively, and thereby avoid solving the Jacobi matrix.

According to the iterative calculation process of IAFSR-UKF, the main differences between this algorithm and the classical UKF algorithm are as follows:

(1) By using Cholesky and QR decomposition, the square root is used instead of the error covariance matrix to participate in the recursive operation. Therefore, the problem that the matrix is easy to fall into negative definite is successfully avoided. Furthermore, the problem of filter divergence is solved and the stability of numerical calculation is also ensured.
(2) According to the theory of the strong tracking filter, a fading factor is introduced to adjust the square root of the filter gain matrix and the output covariance matrix in real-time. When there is a significant error in the equivalent measurement value, the parameter ***C****_t_* increases, and the corresponding adaptive factor increases, resulting in the increase of the square root of the output covariance matrix, thereby reducing the filter gain, and then reducing the impact of measurement noise on the state update. Therefore, compared with the classical UKF, the proposed algorithm has better model mismatch robustness and excellent strong tracking ability.
(3) In addition, the improved calculation method of the fading factor can effectively reduce the computational complexity while ensuring the filtering accuracy.

## 3. Performance Evaluation and Discussion

The multi-sensor combined measurement method developed in this paper was tested through laboratory bench and field measurement data, respectively. Comparison analysis with classical UKF [5] was conducted to comprehensively evaluate the performance of the proposed IAFSR-UKF.

### 3.1. Laboratory Testing

**Figure 2:**
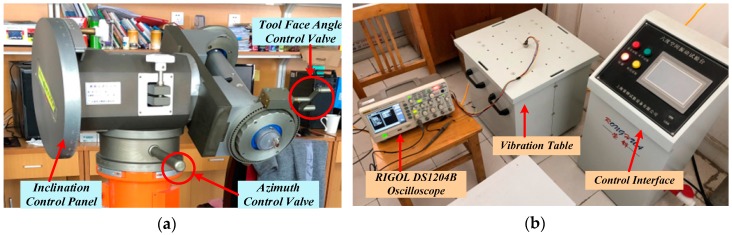
The main instruments and equipment in the laboratory: (**a**) inclinometer adjusting device; (**b**) six-degree space vibration experiment platform and oscilloscope.

The nonlinear filtering algorithm for multi-sensor combined measurement was tested first in a laboratory environment, as shown in Figure 2. The geographical conditions of the laboratory are as follows: 34.21° N, the earth’s rotation speed is 15°/h, the geomagnetic inclination is 55.42°, the magnetic field intensity is 52.7 μT, and the earth rotation velocity is 9.8 m/s^2^. 

The main instruments and equipment for laboratory testing included a set of inclinometer calibration device, whose model was TX-3S, and the six-degree space vibration experimental platform, as shown in Figure 2a,b, respectively. Other selected experimental equipment included RIGOL DS1204B oscilloscope, a data acquisition system, DC power supply, etc.

**Figure 3:**
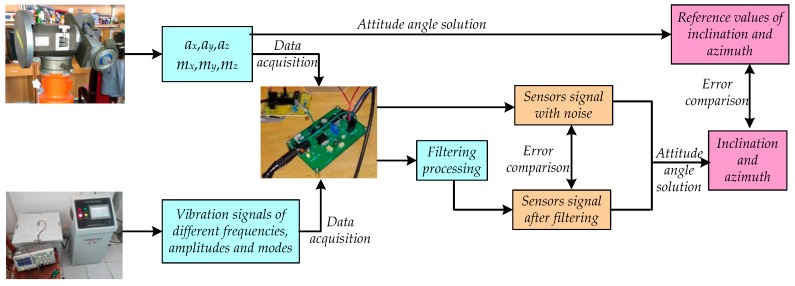
The measurement schematic diagram of laboratory testing.

The measurement schematic diagram is shown in Figure 3. Firstly, the three-axis accelerometer and the three-axis magnetometer sensor were obtained by rotating the control valve in the three directions of the inclinometer; the vibration frequency, amplitude, and vibration mode of the six degree space vibration experimental platform were set to obtain the required vibration signal. Then, the electrical signal was converted to digital and stored in the data acquisition card. The obtained digital signal was processed by classical UKF and the proposed IAFSR-UKF, respectively. Finally, the output signal of the measurement sensor and the attitude angle parameters were compared to verify the effectiveness of the proposed method.

The error characteristics of the triaxial accelerometer and the triaxial magnetometer were the same. The interference measured in the laboratory environment can be approximated to the Gaussian white noise with zero mean. The relevant parameters in the proposed algorithm were set as *α* = 0.06, *β* = 4, and *ρ* = 0.95.

It should be noted that the research object of this article is the data fusion algorithm for multi-sensor combined measurement. The sensor data, which was obtained through uplink communication, can be processed by the control center on the ground. Therefore, the adverse influence of high temperature environment on the algorithm was not discussed in the laboratory testing.

Under the laboratory condition, simulating the server vibration environment in the well, the drilling tool was kept in the rotating state with the angular velocity of 1 rad/s. In the initial 500 groups of the experiment data, the intensity of the vibration signal was set to two times of the useful signal, to the middle 500 groups of data, the vibration intensity was adjusted to five times of the useful signal, and finally, 500 groups of data restored the intensity of the vibration signal to two times of the useful signal to verify the adaptive ability and stability of the proposed algorithm. 

**Figure 4:**
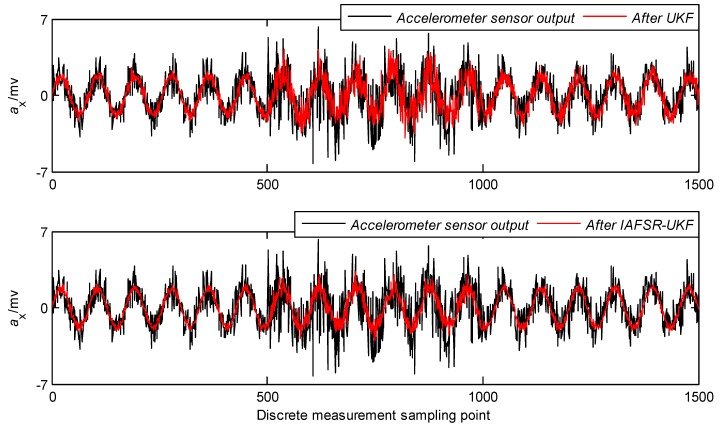
Filtering results of x-axis accelerometer sensor.

**Figure 5:**
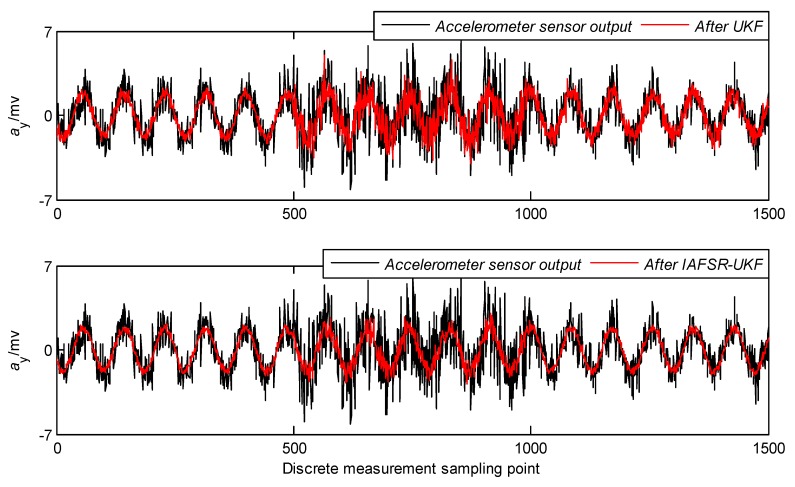
Filtering results of y-axis accelerometer sensor.

**Figure 6:**
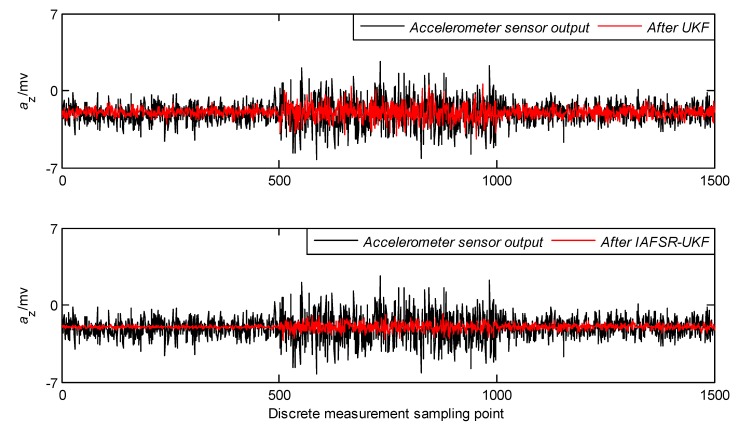
Filtering results of z-axis accelerometer sensor.

**Figure 7:**
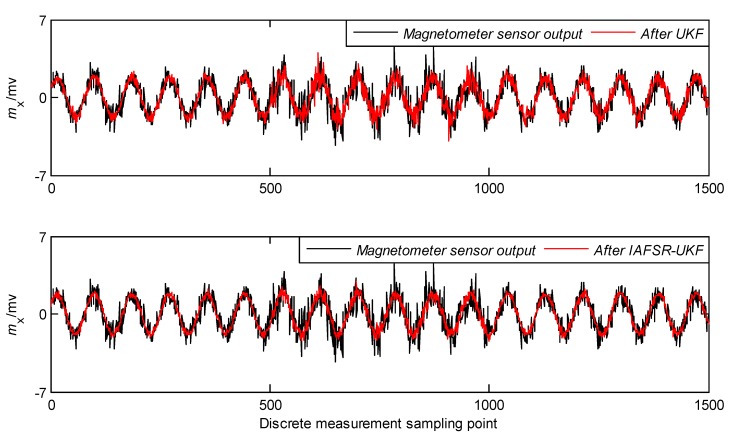
Filtering results of x-axis magnetometer sensor.

**Figure 8:**
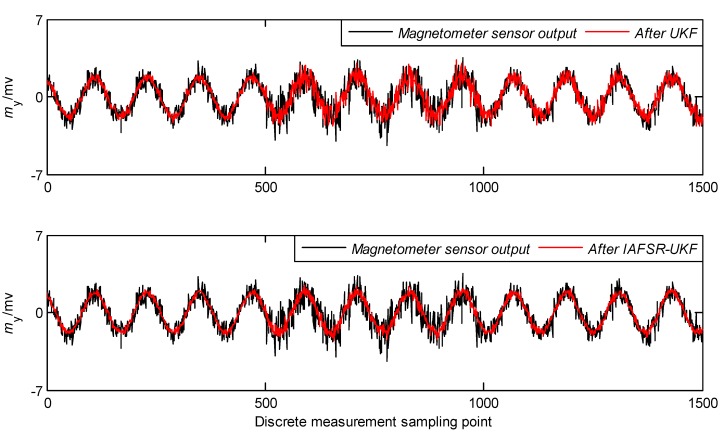
Filtering results of y-axis magnetometer sensor.

**Figure 9:**
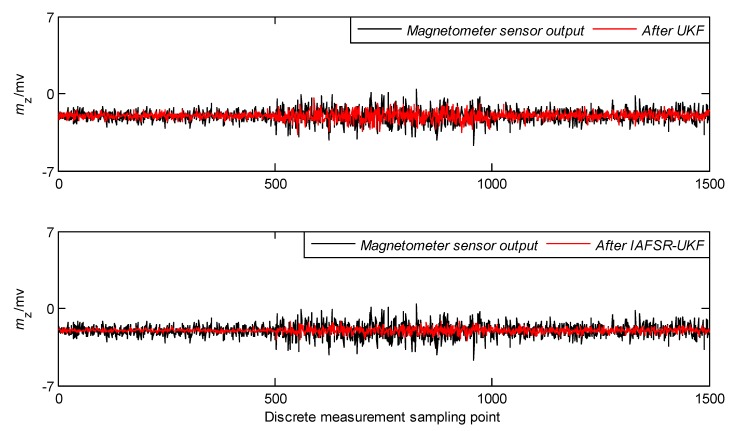
Filtering results of z-axis magnetometer sensor.

In order to verify the performance of the proposed algorithm, under the same experimental conditions, the same experimental data was filtered with classical UKF and IAFSR-UKF. The filtering results of the three-axis accelerometer and the three-axis magnetometer data are shown in Figure 4, Figure 5, Figure 6, Figure 7, Figure 8 and Figure 9.

It can be seen from Figure 4, Figure 5, Figure 6, Figure 7, Figure 8 and Figure 9 that the fluctuation of each measurement sensor after filtering by the IAFSR-UKF is significantly smaller than that of the classical UKF algorithm, which shows that the proposed algorithm has better noise suppression ability. Moreover, the accelerometer noises are relatively much larger than the magnetometer signal noises. The main reason is that accelerometers are hypersensitive to drill string vibrations. In the field test, the vibration of the drill string will be more violent.

It is worth noting that the filtering effect of the classical UKF is poor while the noise intensity suddenly increases at the 500th~1000th sampling point, and the filtered output waveform still contains a strong noise signal. On the contrary, the proposed IAFSR-UKF algorithm has a better filtering effect in which the output waveforms of x-axis and y-axis measurement sensors are close to the sinusoidal signal, showing the better adaptive ability to the sudden change of the measurement environment and excellent calculation stability.

**Figure 10:**
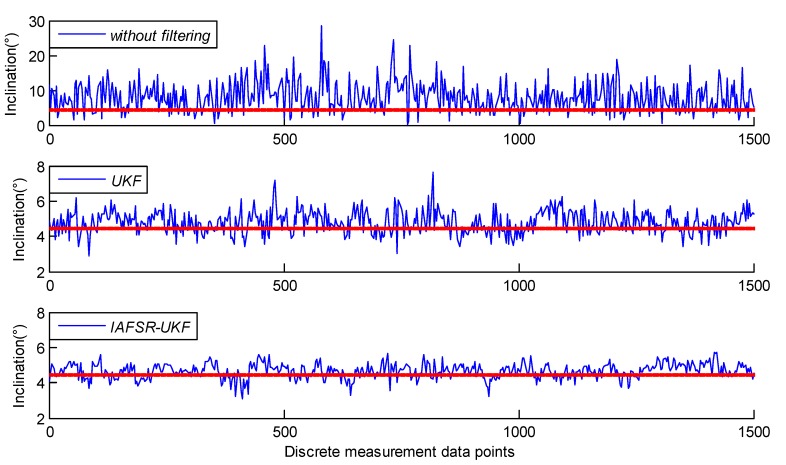
Calculating results of inclination.

**Figure 11:**
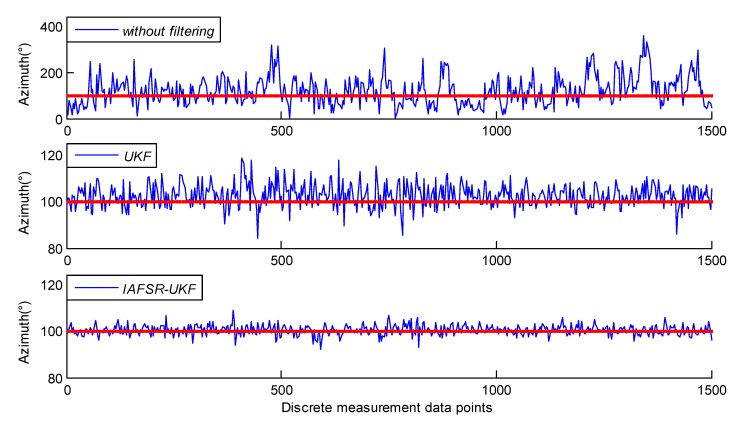
Calculating results of azimuth.

In order to verify the influence of the proposed algorithm on the accuracy of the drilling tool attitude solution, the classical UKF and the IAFSR-UKF were used for attitude solution, respectively, and the simulation results are shown in Figure 10 and Figure 11.

The azimuth is determined by three-axis magnetometers, while the inclination is determined by three-axis accelerometers. Therefore, without filtering, the error of the continuous measurement will be dramatically amplified due to drill string vibration and rotation, as shown in Figure 10 and Figure 11.

It can be seen that the deviation of the inclination and the azimuth value, which were obtained by classical UKF, was more significant. Therefore, it was impossible to accurately judge the drilling tool attitude according to the solution result; especially on the data points with increased noise intensity, the error fluctuation amplitude obviously increased, and the maximum error value exceeded 3° and 18°, respectively.

On the other hand, the deviation of the drilling tool attitude angle calculated by the proposed IAFSR-UKF was obviously smaller, which is more close to the actual attitude parameters. Especially, in the case of a sudden increase in noise amplitude, the attitude calculation result is relatively stable, without noticeable error fluctuation. It can be seen that under the server vibration and noise interference, the proposed algorithm can obtain more accurate and stable attitude calculation parameters; and when the measurement system is abnormal, the proposed algorithm can still effectively track the attitude parameters while drilling.

Further statistics show the detailed error results, as shown in Table 1.

It can be seen from Table 1 that the root mean square (RMS) error of the inclination and the azimuth was only 0.46° and 2.18°, respectively after IAFSR-UKF. It is not only far less than the attitude calculation error of the original measured value, but also better than the solution result of the classical UKF. Furthermore, the feasibility and validity of the proposed algorithm was proved in laboratory testing.

### 3.2. Field-Drilling Testing

**Figure 12:**
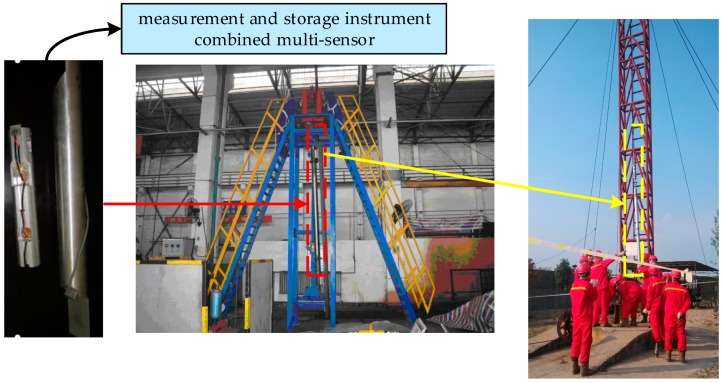
Schematic of the field test. The electronic circuit was installed in the axis of the drill collar near-bit, and the measurement data can be stored in real-time.

In order to further verify the feasibility and overall performance of the proposed multi-sensor data fusion algorithm, field-drilling data were used for test and analysis. The experimental data came from the field-drilling process of a well in western Sichuan from 20 November to 24 November, 2014. The field acquisition process and installation position of the triaxial sensor are shown in Figure 12, and the drilling and production environment parameters are listed in Table 2. During drilling, the guiding tool was in a stable and straight state.

The HMC5983 high-precision sensor of the Honeywell Company was selected as the magnetometer in a field-drilling test, and the CS-3LAS sensor, which was developed by the Zhongxing measurement and control company, was chosen as the accelerometer in this test. The accelerometer is suitable for the specific requirements of downhole drilling. The specific parameters of the sensors are shown in Table 3.

**Figure 13:**
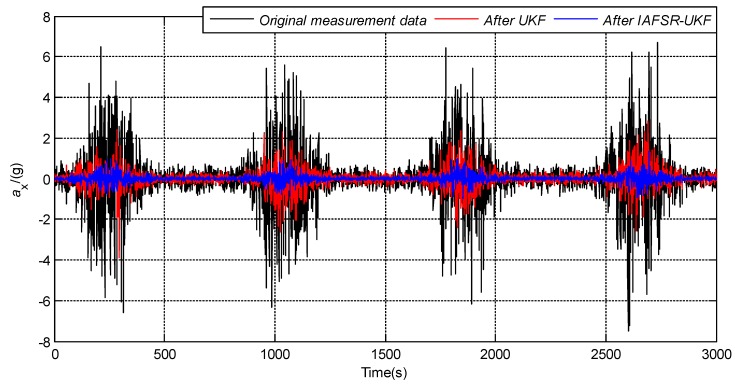
Filtering results of the field-drilling signal of x-axis accelerometer sensor.

**Figure 14:**
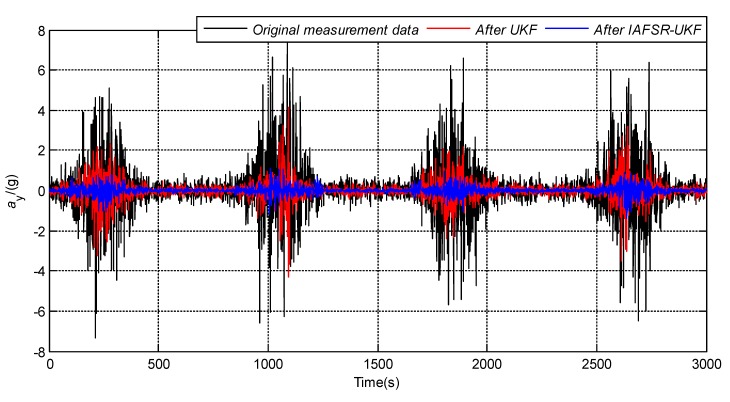
Filtering results of the field-drilling signal of y-axis accelerometer sensor.

**Figure 15:**
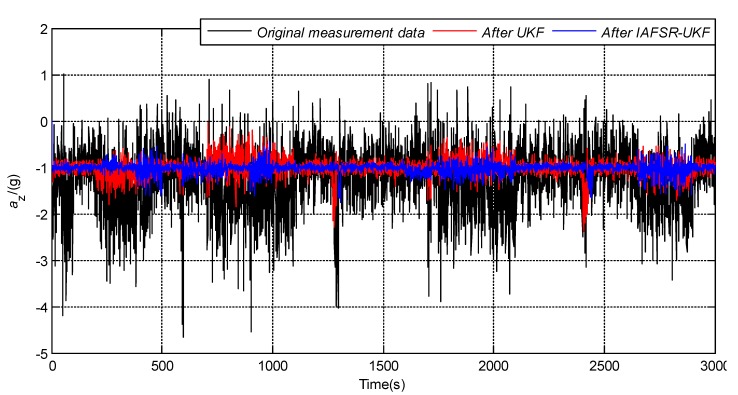
Filtering results of the field-drilling signal of z-axis accelerometer sensor.

Figure 12 shows the schematic of the field test. From the measurement data stored in real-time, we can obtain the vibration signals measured by the accelerometer (x, y, and z axes), which contain the gravity acceleration on the x, y, and z axes. We used the real field-test data to demonstrate the feasibility of our algorithm. The comparison results are shown in Figure 13, Figure 14 and Figure 15. It can be seen that the accelerometer signals of the field tests are entirely different from those obtained via our laboratory survey. We cannot estimate the error of this process, because the data came from the field test where exact measurements are impossible to determine. However, it is evident that the fluctuation amplitude of each accelerometer sensor data, which was processed by IAFSR-UKF algorithm, was significantly smaller. It showed better noise anti-interference ability.

To further evaluate the overall performance of the proposed algorithm, the data after filter processed were solved to obtain the real-time drilling tool attitude parameters. There was no interference of vibration acceleration in the attitude measurement of stopping drilling, which can ensure the accuracy of azimuth and inclination. Therefore, it was used as a reference value to verify the performance of the proposed algorithm. The attitude calculation results and static measurement results obtained after filtering by two filter algorithms during rotary steering drilling are shown in Table 4.

It can be seen from Table 4 that the overall solution accuracy of field-drilling data is lower than that of simulation data, especially the inclination. This is mainly because of the process of drilling, where the measured signal is interfered by many factors, including server vibration, drill string rotation, high temperature, pressure, etc. On the other hand, vibration interference is not an ideal Gaussian white noise signal in the process of drilling. All of these will affect the result of the filtering. In addition, the inclination is more affected by the accelerometer data, and the accelerometer sensor is more affected by the vibration interference than the magnetometer. Therefore, the deviation error of inclination is more significant than that of azimuth.

It can also be concluded from Table 4 that the relative error of the drilling tool inclination after filtering by the proposed algorithm is within 20.8% and the relative error of azimuth is within 5.34%. Compared with classical UKF, the accuracy of attitude solution is obviously improved, and the error fluctuation amplitude is significantly reduced. This shows that the proposed IAFSR-UKF in this paper provides a new solution to the problem of multi-sensor continuous MWD.

### 3.3. Real-Time Evaluation

In order to further verify the advantages of the IAFSR-UKF algorithm in real-time, the time required for each simulation calculation with the UKF, AFSR-UKF, and IAFSR-UKF algorithms are recorded, respectively, and the average time required for 10 filtering calculations is taken as the real-time statistical results. The average filtering time of UKF, AFSR-UKF, and IAFSR-UKF are represented by *t_UKF_*, *t_AFSR_*_,_ and *t_IAFSR_*, respectively. Based on AFSR-UKF, a set of relative execution time is defined as follows:

(41) $$t_{UKF}^{R}=\frac{t_{UKF}}{t_{AFSR}},\;t_{ASFR}^{R}=\frac{t_{AFSR}}{t_{AFSR}},\;t_{IAFSR}^{R}=\frac{t_{IAFSR}}{t_{AFSR}}.$$

**Figure 16:**
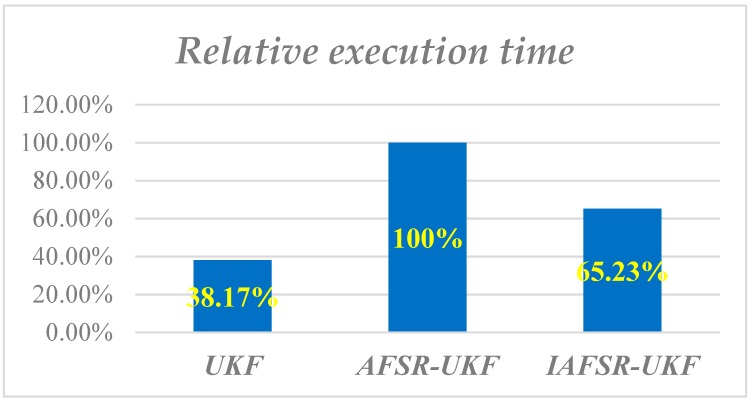
The relative execution time of the three algorithms for multi-sensor data fusion.

The relative execution time of the three algorithms is shown in Figure 16.

As can be seen from Figure 16, the execution time of a one-time filtering calculation with UKF was the shortest, only 38.17% of the AFSR-UKF. This is because the classical UKF does not need to calculate the fading factor and the square root of the covariance matrix. Compared with the AFSR-UKF, the improved fading factor calculation method also showed its superiority in execution time, saving about 35% of calculation time. This is because the IAFSR-UKF does not need the Jacobi expansion of the state equation and the measurement equation in calculating the fading factor, which reduces the computational complexity.

In addition, according to the statistics of the filtering time of the field-drilling data, the single filtering and attitude solution time with AFSR-UKF was close to 0.8 s, while the single filtering and solution time with the proposed IAFSR-UKF was less than 0.5 s. Therefore, the improved fade factor calculation method can greatly improve the calculation efficiency of the algorithm and meet the real-time requirements of MWD.

## 4. Conclusions

This paper presents an IAFSR-UKF algorithm to improve the adaptability and numerical stability for multi-sensor combined MWD. The contributions of this paper are (i) through the simulation test of the vibration platform system in the laboratory and the field-drilling experiment. It can be seen that the proposed IAFSR-UKF algorithm can effectively reduce the impact of the server vibration of the near bit on the dynamic MWD, improve the measurement accuracy of the original signal of the sensors, and then improve the accuracy of the attitude solution, which verifies the feasibility of the proposed method; (ii) compared with the classical UKF, the error fluctuation amplitude of sensors which were processed by the proposed algorithm is obviously reduced. It is proved that the proposed algorithm has better computational stability; on the other hand, in the face of the sudden change of noise intensity, the proposed algorithm has better data fusion performance, which shows that it can effectively improve the adaptive ability of the mutation state; and (iii) the improved calculation method of fade factor can effectively reduce the calculation complexity and computational time-consuming so that the algorithm can meet the real-time requirements of MWD.

Future research studies will focus on two aspects. One is the improvement of the proposed IAFSR-UKF. It is expected to combine the IAFSR-UKF with spectrum estimation, array signal processing, and other methods, which provides a new idea for the multi-sensor continuous MWD. On the other hand, according to the complex characteristics of the interference noise during the field-drilling under the well, how to improve the comprehensive processing ability of the proposed algorithm is another problem to be solved in future research studies.

## Figures and Tables

**Table 1:** Error statistics of attitude solution after filtering.

Algorithm	Drilling Tool Attitude Angle	Statistics of Filtering Solution Error	
		Max Error	RMS Error
IAFSR-UKF	inclination (°)	1.42	0.46
	azimuth (°)	8.78	2.18
Classical UKF	inclination (°)	3.11	0.72
	azimuth (°)	18.61	5.47
Without filtering	inclination (°)	24.01	5.54
	azimuth (°)	257.57	65.17

**Table 2:** Basic parameters of the field-drilling test.

Parameter	Value
Well depth	1740–1807 m
WOB	10 MPa
Downhole temperature	40 °C
Pump pressure	6.6 MPa
Drilling fluid density	1.15 g/cm^3^
Suspended load	79 kN
Operation time	75 h
Rotary speed	120 rpm
The setting value of inclination	2.5°

**Table 3:** Characteristics of sensors.

Parameter	Accelerometer (CS-3LAS)	Magnetometer (HMC5983)
Range	±10 g	±100 μT
Scale factor	200 mv/g	5 V/G ± 5%
Non-linearity	0.8%	-
Calibration	<50 mg	±0.005 G
Noise	0.14 mg/Hz^1/2^	≤0.2 nT
Bandwidth	1000 Hz	300 Hz

**Table 4:** Comparison of attitude calculation after filtering with static measurement results.

Algorithm	Attitude Parameters	Depth/m				
		1754.265	1765.36	1776.455	1787.55	1798.645
Static measurement	Inclination (°)	2.39	2.52	2.50	2.42	2.47
	Azimuth (°)	200.8	205.5	215.2	202.4	211.8
UKF	Inclination (°)	2.17	1.90	3.46	3.11	3.22
	Relative error	9.2%	24.6%	38.4%	28.5%	30.4%
	Azimuth (°)	181.0	211.1	201.4	189.5	223.5
	Relative error	9.86%	2.73%	6.41%	6.37%	5.52%
MSTSR-UKF	Inclination (°)	2.78	2.90	3.02	2.16	2.94
	Relative error	16.3%	15.1%	20.8%	10.7%	19%
	Azimuth (°)	204.3	209.8	203.7	208.3	204.5
	Relative error	1.74%	2.09%	5.34%	2.92%	3.45%
